# Comparative genomic analysis of *Myroides odoratimimus* isolates

**DOI:** 10.1002/mbo3.634

**Published:** 2018-05-23

**Authors:** Shaohua Hu, Lin Cao, Yiyin Wu, Yajun Zhou, Tao Jiang, Liqiang Wang, Qiujing Wang, Desong Ming, Shicheng Chen, Mingxi Wang

**Affiliations:** ^1^ State Key Laboratory for Diagnosis and Treatment of Infectious Diseases Collaborative Innovation Center for Diagnosis and Treatment of Infectious Diseases The First Affiliated Hospital of Medical College Zhejiang University Hangzhou Zhejiang China; ^2^ School of Medicine and School of Biomedical Sciences Huaqiao University Xiamen Fujian China; ^3^ College of Computer Science and Technology Huaqiao University Xiamen Fujian China; ^4^ Department of Neurosurgery Shenzhen Hospital Southern Medical University Shenzhen, Guangdong China; ^5^ Department of Clinical Laboratory Quanzhou First Hospital Affiliated to Fujian Medical University Fujian China; ^6^ Department of Microbiology and Molecular Genetics Michigan State University East Lansing MI USA

**Keywords:** antibiotic resistance genes, comparative genomics, *Myroides odoratimimus*, virulence factors

## Abstract

*Myroides odoratimimus* is an important nosocomial pathogen. Management of *M. odoratimimus* infection is difficult owing to the multidrug resistance and the unknown pathogenesis mechanisms. Based on our previous genomic sequencing data of *M. odoratimimus* PR63039 (isolated from a patient with the urinary tract infection), in this study, we further performed comparative genomic analysis for 10 selected *Myroides* strains. Our results showed that these *Myroides* genome contexts were very similar and phylogenetically related. Various prophages were identified in the four clinical isolate genomes, which possibly contributed to the genome evolution among the *Myroides* strains. CRISPR elements were only detected in the two clinical (PR63039 and CCUG10230) isolates and two environmental (CCUG12700 and H1bi) strains. With more stringent cutoff parameters in CARD analysis, the four clinical *M. odoratimimus* contained roughly equal antibiotic resistance genes, indicating their similar antibiotic resistance profiles. The three clinical (CCUG10230, CCUG12901, CIP101113) and three environmental (CCUG12700, L41, H1bi) *M. odoratimimus* strains were speculated to carry the indistinguishable virulent factors (VFs), which may involve in the similar pathogenesis mechanism. Moreover, some VFs might confer to the high capacity of dissemination, attacking tissue cells and induction of autoimmune complications. Our results facilitate the research of antibiotic resistance and the development of therapeutic regimens for the *M. odoratimimus* infections.

## 1 INTRODUCTION

*Myroides odoratimimus* is a gram‐negative and opportunistic pathogen. It causes a variety of serious infections mainly reported in China (summarized by Hu et al., 2016) and outbreak of urinary tract infection (Ktari et al., 2012). Recently, the increasing infections emerged in patients with the recurrent calcaneal ulcer (Pompilio et al., 2017), fulminant erysipelas and sepsis (Willems, Muller, Verhaegen, Saegeman, & Desmet, 2017), bacteremia (Belloir, Billy, Hentgen, Fille, & Barrans, 2016), or prosthesis joint infection (Jover‐Sáenz, Pérez‐Villar, & Barcenilla‐Gaite, 2016).

*M. odoratimimus* infections are life‐threatening due to its multidrug resistance and unknown pathogenicity (as summarized in Hu et al., 2016). In our previous report (Hu et al., 2017), to some extent, we correlated the phenotype in antibiotic susceptibilities and infectivity of *M. odoratimimus* with the genomic findings of a variety of resistance genes, virulence factor (VF) genes. To better accomplish these purposes and verify the possibility of infection source of environmental strains, here, we further performed comparative genomic analysis of 10 *Myroides* strains, including four clinically pathogenic (PR63039, CCUG10230, CCUG12901, CIP101113), four environmental (CCUG 3837 CCUG 12700 H1bi L41), and two human‐associated *Myroides* isolates (CCUG39352, *Myroides* sp. A21) by focusing on their antibiotic resistance and pathogenesis mechanisms.

## 2 MATERIALS AND METHODS

### 2.1 Genome sequences

In the NCBI Genome RefSeq Assembly Database, only nine genomic sequences of *M. odoratimimus* were found (Table 1). They included four clinically pathogenic strains, a human‐associated strain, and four environmental isolates. Only PR63039 genome (Hu et al., 2017) was complete. Strain PR63039 (Hu et al., 2017) and CCUG12901 were isolated from the urine of patients with postinjury urinary tract infection. CCUG10230 and CIP101113 were isolated from skin wounds. Human‐associated strain CCUG39352 was collected and sequenced by Shandong University. *M*. *odoratimimus* H1 bi, L41, CCUG 12700, and CCUG 3837 are environmental isolates. For the phylogenetic tree analysis of *Myroides* genomes, another human‐associated strain *Myroides* sp. A21 (CP010327) (Burghartz et al., 2015) with highly homologous 16S rRNA gene sequence to strain PR63039 (coverage 100%, identity 100%, 1,388 bp) (GenBank No. KR349266) was also included. *Myroides* sp. A21 was isolated from the urethral catheter of a patient without symptoms of a urinary tract infection, had extensive drug resistance; its full genomic sequence was available.

**Table 1:** General genomic characteristics of 10 *Myroides* strains

Sources	Strain	Site of isolation	Type	Assembly No.	Level	Scaffold	Size (Mb)	GC (%)	Gene	Protein	rRNA	tRNA	Other RNA	Pseudo gene
Clinically pathogenic *M. odoratimimus*	PR63039	Urine		GCA_001481655.1		2	4.46	34.14	4,084	3,840	27	105	4	108
			Chr	NZ_CP013690.1	Complete		4.37	34.20	3,988	3,745	27	105	4	107
			Plsm	NZ_CP013691.1			0.09	31.30	96	95	‐	‐	‐	1
	CCUG 10230	Skin wound	Chr	GCA_000242075.2	Scaffold	7	4.03	34.10	3,610	3,458	5	76	4	67
	CCUG 12901	Urogenital tract	Chr	GCA_000242095.1	Scaffold	3	4.07	34.20	3,653	3,505	9	75	4	60
	CIP 101113	Skin wound	Chr	GCA_000242135.1	Scaffold	3	4.14	34.10	3,673	3,516	8	71	4	74
Environmental *M. odoratimimus*	CCUG 3837	N/A	Chr	GCA_000297855.1	Scaffold	4	4.14	34.40	3,611	3,459	11	72	4	65
	CCUG 12700	N/A	Chr	GCA_000413415.1	Scaffold	7	4.04	34.30	3,581	3,423	7	87	4	60
	H1bi	*Carnivorous plant Phytotelma*	Chr	GCA_000633375.1	Contig	183	3.88	34.00	3,549	3,260	4	85	4	196
	L41	Lake water	Chr	GCA_000812825.1	Contig	168	4.16	33.60	3,802	3,599		82	4	114
Human‐associated *Myroides*	CCUG 39352	Wound	Chr	GCA_001485415.1	Contig	65	4.24	33.90	3,789	3,650		74	‐	‐
	*Myroides sp*. A21	Urethral catheter	Chr	GCA_000807225.1	Complete	1	4.16	34.10	3,750	3,554	24	101	4	67

Chr, chromosome; Plsm, plasmid; N/A, not available.

### 2.2 Softwares and databases used for comparative genomics analysis

The analyses of whole‐genome phylogenetic tree, circular genome mapping, insertion sequence elements (IS), multiple genome alignment, prophage, CRISPR, antibiotic resistance genes, and VF genes in the *M. odoratimimus* genomes were performed with the softwares and databases listed in Table 2.

**Table 2:** The softwares and databases used for comparative analysis

Analysis	Software/database	References	Clinically pathogenic strains				Environmental strains				Human‐associated strains	
			PR63039	CCUG 10230	CCUG 12901	CIP 101113	CCUG 12700	L41	H1bi	CCUG 3837	CCUG 39352	*Myroides sp*. A21
Sequence level			Complete	Scaffold	Scaffold	Scaffold	Scaffold	Contig	Contig	Scaffold	Contig	Complete
Phylogenetic tree	REALPHY	Bertels, Silander, Pachkov, Rainey, & van Nimwegen, 2014;	Yes	Yes	Yes	Yes	Yes	Yes	Yes	Yes	Yes	Yes
CG	CG viewer	Grant & Stothard, 2008;	Yes	Yes	Yes	Yes						
ISs	IS Finder	Siguier, Perochon, Lestrade, Mahillon, & Chandler, 2006;	Yes (43)	Yes (169)	Yes (117)	Yes (104)						
Synteny	Progressive mauve	Darling et al., 2010;	Yes	Yes	Yes	Yes	Yes	Yes	Yes			
Prophage	PHAST	Zhou, Liang, Lynch, Dennis, & Wishart, 2011;	Yes (2 incomplete)	Yes (2)	Yes (5)	Yes (1)						
CRISPR	CRISPR Finder	Bland et al., 2007;	Yes (3)	Yes (4)	Yes (ND)	Yes (ND)	Yes (1)	Yes (ND)	Yes (1)			
Antibiotic resistance genes	CARD Resistance Gene identifier	Mcarthur et al., 2013; Jia et al., 2017;	Yes	Yes	Yes	Yes						
Virulence factors	VFDB protein Set B database	Chen et al., 2012, 2016		Yes	Yes	Yes	Yes	Yes	Yes			

CG, Circular genome maps (Genomic variants); ISs, insertion sequence elements; Yes, analyzed; (): number of the predicted; ND, not detected.

We should mention that, for identifying the resistance genes using CARD Resistance Gene Identifier (RGI) software (Jia et al., 2017; Mcarthur et al., 2013), we performed BLASTp search (collaborated with Beijing Novogene Bioinformatics Technology Co., Ltd, BNNT) of the protein sequences of *M. odoratimimus* (downloaded from RefSeq assembly) against the CARD reference sequences, and more stringent parameters were set up [Query ID, Chromosome, Gene start, Gene end, Direction, ARO ID, ARO name, Category, Query length, Query start, Query end, Subject length, Subject start, Subject end, Gap, Mismatch length, Match length, Bit score, E value ≤ 1e‐30, Identity (%), Query coverage, Subject coverage]. The stringency of extracting antibiotic resistance genes from the primary output was improved by setting the cutoff parameters (Protein identity [>50%], Query coverage [>50%], and Subject coverage [>50%]).

The genes coding for the virulence factors was predicted by performing BLAST search (collaborated with Beijing Novogene Bioinformatics Technology Co., Ltd) of the protein sequences of *M. odoratimimus* against the VFDB protein Set B database (Chen, Xiong, Sun, Yang, & Jin, 2012; Chen, Zheng, Liu, Yang, & Jin, 2016). The stringent parameters were set up (Gene ID, VFDB internal ID, VF ID, VF name, Genes, Characteristics, Structure features, Functions, Mechanisms, Descriptions, Query length, Query start, Query end, Subject length, Subject start, Subject end, Match length, Mismatch length, Gap, Identity, E value, Bit score, Query coverage, Subject coverage). The cutoff parameters for extracting VF genes from the primary outputs were same as the extracting resistance genes as the above.

## 3 RESULTS

### 3.1 The basic genome statistics of 10 *Myroides* genomes

The general features of 10 *Myroides* genomes**,** including four clinically pathogenic *M. odoratimim*us (PR63039, CCUG12901, CCUG10230, CIP101113), four environmental (CCUG 12700, L41, H1bi, CCUG 3837), and two human‐associated *Myroides* strains (CCUG 39352, *Myroides sp*. A21) were similar (summarized in Table 1). Their GC contents were approximately 34%. The sizes of the genomes varied from 3.88 to 4.46 Mb. The numbers of genes, proteins, and tRNAs in PR63039 genome were larger than that of the other nine genomes. We should mention that eight *Myroides* genomes were incomplete, and the sequencing of the plasmids in PR63039 strains were not completed even its chromosome was fully sequenced (Figure 1 in Hu et al., 2016, 2017).

### 3.2 Phylogenetic analysis of 10 *Myroides* genomes

**Figure 1:**
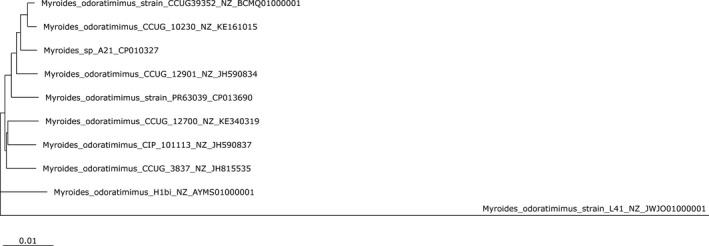
Whole‐genome phylogenetic tree of 10 *Myroides* isolates. This Whole‐genome phylogenetic tree was produced by REALPHY with the default parameters. Strain L41 was used as the root

Whole‐genome phylogenetic tree of the 10 *Myroides* genomes was created (Figure 1). It showed that PR63039 formed a different clade from *Myroides* sp. A21 even although they had highly homologous 16S rRNA gene sequence (100% identity). The clinically pathogenic strain CCUG 10230 and the environmental strain CCUG 39352 were closest. Strain CCUG3837, CIP101113, and CCUG12700 formed a clade, while strain H1bi was located alone. It seemed that strain CCUG3837, CIP101113, and CCUG12700 were phylogenetically closer and H1bi might have a different origin compared to the above three strains.

Collectively, these 10 *Myroides* strains were related phylogenetically, indicating that there might similarly evolve to some pathogenesis traits. However, further study to illuminate the evolution pathway is warranted.

### 3.3 Genomic variants among four clinically pathogenic *M. odoratimimus* strains

**Figure 2:**
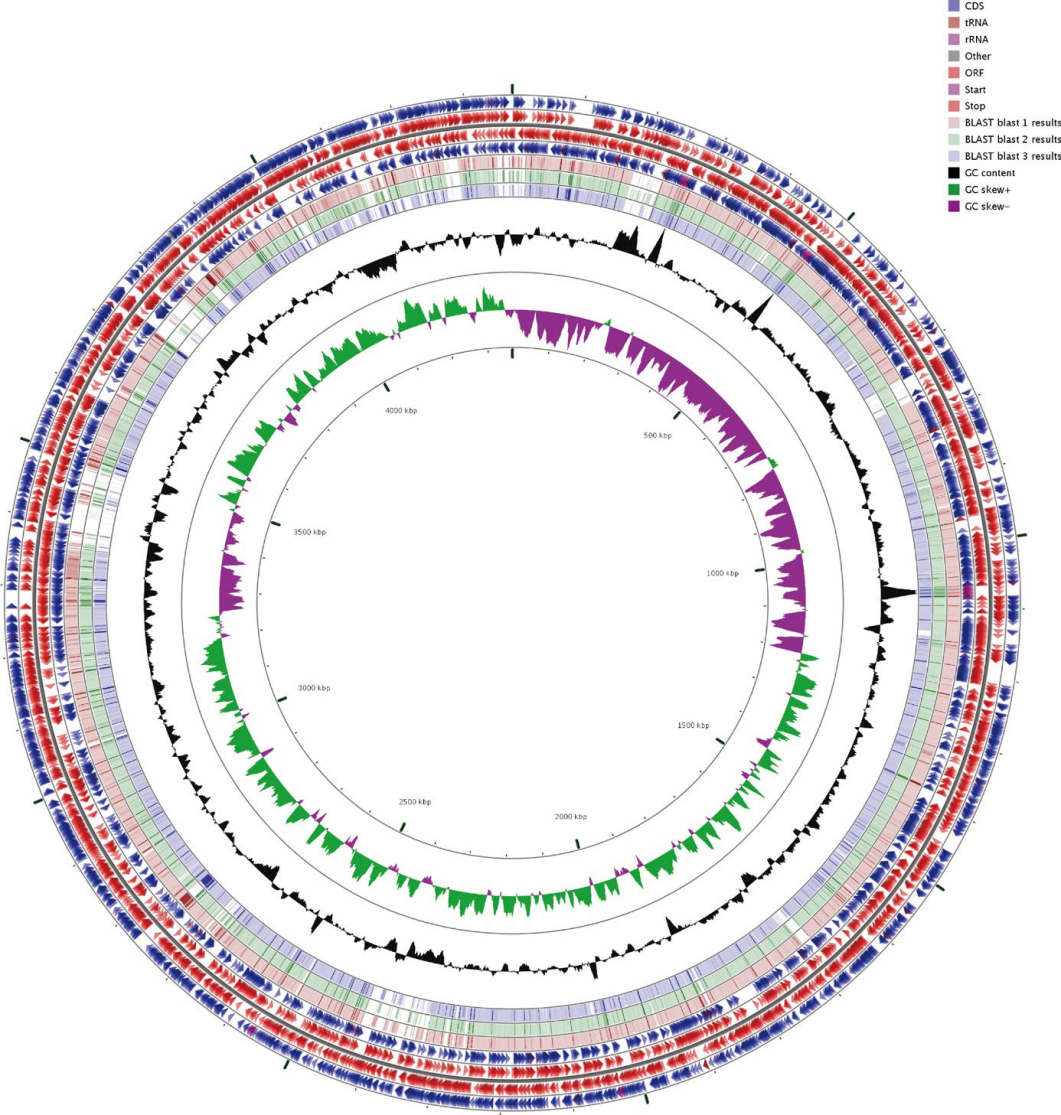
Genomic comparisons in four clinically pathogenic *M. odoratimimus* strains. Blast 1: CCUG10230. Blast 2: *M*. CCUG12901. Blast 3: CIP101113. The contents of the feature rings (starting with the outermost ring) are as follows: Ring 1: forward strand features the read from the primary sequence of PR63039. Rings 2: forward strand ORFs from the primary sequence of PR63039. Rings 3: reverse strand ORFs from the primary sequence of PR63039. Ring 4: reverse strand features read from the primary sequence of PR63039. Ring 5 (vermilion): blast 1. Ring 6 (green): blast 2. Ring 7 (blue): blast 3. Ring 8 (black): the G + C content. Ring 9 (green and purple): the GC skew value

We compared the genomes of three clinically pathogenic *M. odoratimimus strains* (CCUG12901, CCUG10230, CIP101113) with the clinically pathogenic PR63039 genome as the reference (Table 2). Many highly variable regions were found (Figure 2). Specifically, the following regions on the above three genomes were absent or had low identity with our strain PR63039: from 150 to 250 kb, 700 to 780 kb, 1,650 to 1,700 kb, 2,300 to 2,450 kb, 2,680 to 2,720 kb, 3,370 to 3,530 kb, 3,720 to 3,800 kb, 4,110 to 4,200 kb, 4,250 to 4,270 kb, and 4,350 to 4,560 kb. Interestingly, the region from 1,650 to 1,700 kb was predicted to be located in one prophage locus of CCUG‐12901 genome. Circular map of the genome comparisons indicated that there were a number of conserved or diverged genome segments among the genomes of these four clinically pathogenic *M. odoratimimus*.

In addition, the abovementioned variable regions were partially accompanied by several insertion elements, which might assist the integration of resistance‐ and pathogenesis‐related genes and facilitate the transfer of drug resistance and pathogenic genes among *M. odoratimimus* strains. Furthermore, IS elements may enhance drug resistance and virulence by promoting gene expression (Heritier, Poirel, & Nordmann, 2006; Higgins, Dammhayn, Hackel, & Seifert, 2010).

### 3.4 Synteny analysis among four clinically pathogenic and three environmental *M. odoratimimus* strains

Genome alignments can identify evolutionary traits. To study the genome synteny and rearrangements in four clinically pathogenic (PR63039, CCUG10230, CCUG12901, and CIP101113) and three environmental (CCUG12700, L41, and H1bi) *M. odoratimimus* bacteria (Table 2), the genome alignment software progressive MAUVE (Darling, Mau, & Perna, 2010) was used Figure (S1). The synteny between the PR63039 genome and *Myroides* sp. A21 was approximately 83.7% (Hu et al., 2017). The genome arrangement of these four clinically pathogenic isolates mimics each other. Similarly, the genome context and arrangement in the three environmental strains exhibited great similarity. However, the genome synteny between the clinically pathogenic and environmental isolates was relatively low.

The alignment of the four genomes of clinically pathogenic isolates showed that their genome rearrangements were similar although there were inversions in some regions. Moreover, the chromosomal alignments of CCUG12901 and CIP101113 were nearly identical with large segments of high similarity. There were some white areas not aligned well because they might contain elements specific to a particular genome.

Overall, the four clinically pathogenic strain genomes were similar although the genome synteny in the latter three (CCUG10230, CCUG12901, and CIP101113) was more related than that in PR63039.

### 3.5 Prophages in four clinically pathogenic *M. odoratimimus* strains

**Figure 3:**
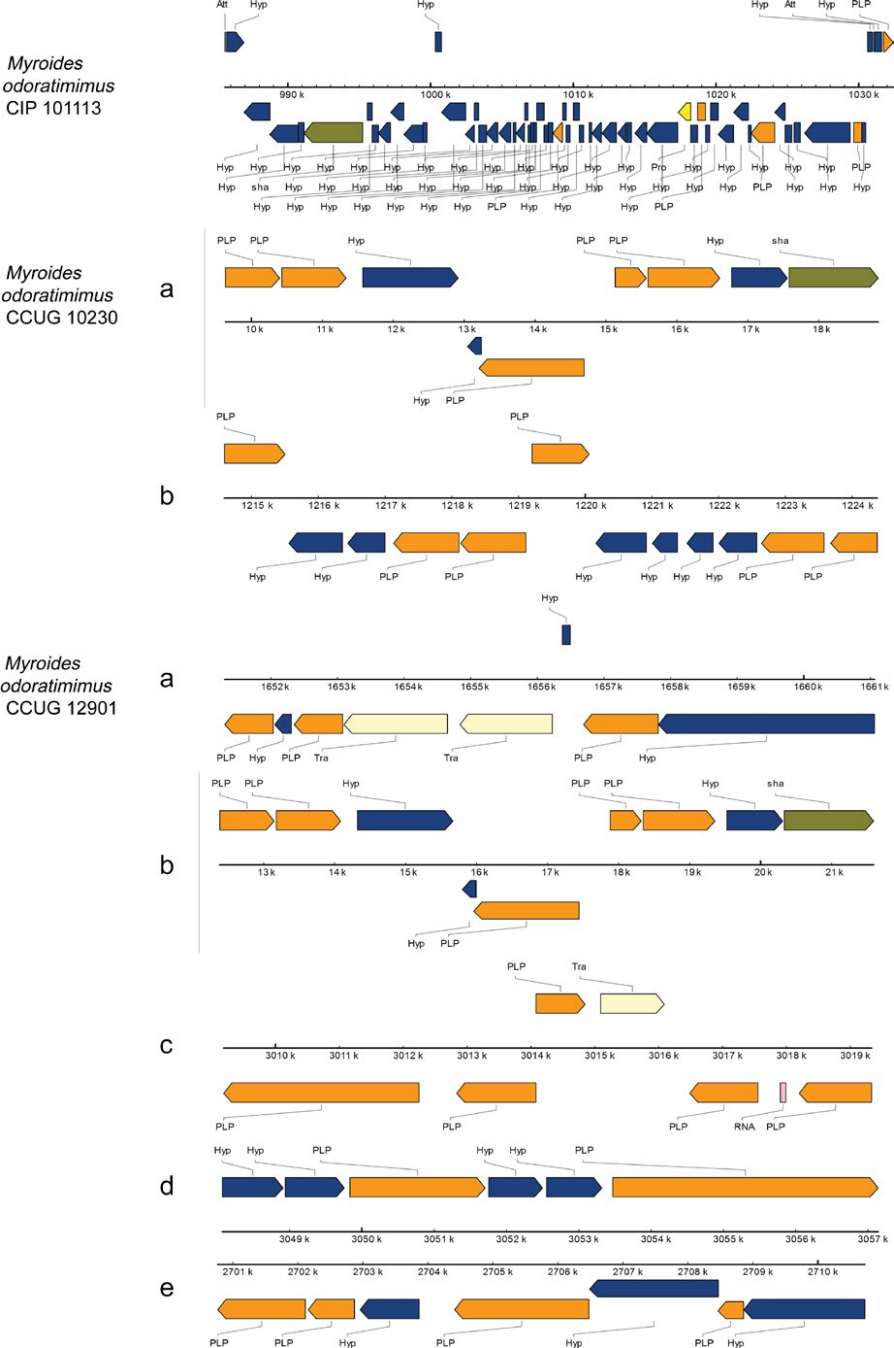
Prophage regions and predicted elements in three clinically pathogenic *M. odoratimimus* strains. Different colored rectangles indicated different phage elements. Att, attachment site; Hyp, hypothetical protein; PLP, Other phage‐like protein; sha, Tail shaft; Pro, protease; Tra, transposase

All the four clinically pathogenic isolates genomes contained incomplete prophage elements (Figure 3). In our strain PR 63039, two incomplete prophages were identified (Hu et al., 2017). CIP101113 contained one prophage with 54 CDs extending from 985,588 bp to 1,032,415 bp (46.8 kb). CCUG10230 was predicted to carry two prophages (9 CDs, 9.2 kb and 12 CDs, respectively). CCUG12901 had five prophages. The region length was 9.2, 9.7, 9.9, 10.1, and 9 kb, respectively, and the number of CDs was 9, 8, 7, 6, 6, respectively. CIP101113 contained only one prophage, but it was larger and more complete than any other prophages. It consisted of hypothetical proteins, phage‐like proteins, attachment sites, tail shafts, and proteases. Among these predicted prophages, attachment sites and proteases only existed in CIP101113 prophage. In bacterial genomes, integrases are useful markers for mobile DNA elements, such as prophages, integrative plasmids, and pathogenicity islands (Liu et al., 2015). However, no integrase was identified in these predicted prophages.

### 3.6 CRISPR prediction in the genomes of four clinically pathogenic and three environmental *M. odoratimimus* strains

CRISPR is well known to contribute to the antibiotic resistance and prevent the foreign virulence genes from invading into pathogens. It may be involved in the bacterial evolution, regulation of virulence gene expression, and the enhancement of pathogenicity (Hatoum‐Aslan & Marraffini, 2014). Particularly, in the pathogen, CRISPR is able to edit genome and modulate gene functions as an adaptive immune system (Arras et al., 2016; Sontheimer & Barrangou, 2015). Comparative analysis of the seven strain genomes (PR63039, CCUG10230, CCUG12901, CIP101113, CCUG12700, L41, and H1bi) showed that PR63039 genome contained three types of CRISPRs (Table S1); CCUG10230 genome contained four types of CRISPRs. CCUG12700 and H1bi contained only one CRISPR. However, no CRISPRs were identified in the genomes of CCUG12901, CIP101113, and L41 (Table S1).

### 3.7 Comparative analysis of antibiotic resistance genes in the genomes of four clinically pathogenic *M. odoratimimus* strains

With CARD RGI software (Jia et al., 2017; Mcarthur et al., 2013), all the genomes of four clinically pathogenic *M. odoratimimus* strains PR63039, CCUG10230, CCUG12901, and CIP101113 were predicted to contain a number of genes related to antibiotic resistance, including the β‐lactam resistance gene, fluoroquinolone resistance gene, antibiotic target replacement protein, antibiotic inactivation enzyme, triclosan resistance gene, diaminopyrimidine resistance gene, phenicol resistance gene, elfamycin resistance gene, and efflux pumps conferring antibiotic resistance (Table 3).

**Table 3:** The predicted resistance genes in the genomes of four clinically pathogenic *M. odoratimimus* strains

Category	PR63039	CCUG12901	CCUG10230	CIP101113
Efflux pump complex or subunit conferring antibiotic resistance	*abeS*	*abeS*	*abeS*	*abeS*
	*KPC‐2*	*KPC‐2*	*KPC‐2*	*KPC‐2*
	*‐*	*mefC*	*‐*	*‐*
	*msrB*	*msrB*	*msrB*	*msrB*
	*qacH*	*qacH*	*qacH*	*qacH*
	*rosA*	*rosA*	*rosA*	*rosA*
Determinant of elfamycin resistance	*basS*	*basS*	*basS*	*basS*
	*LpxC*	*LpxC*	*LpxC*	*LpxC*
	*SPM‐1*	*SPM‐1*	*SPM‐1*	*SPM‐1*
Determinant of phenicol resistance	*catB2*	*‐*	*‐*	*‐*
	*catB6*	*‐*	*‐*	*‐*
	*catB7*	*‐*	*‐*	*catB7*
	*catB8*	*‐*	*‐*	*‐*
	*catB9*	*‐*	*‐*	*‐*
	*catB10*	*‐*	*‐*	*‐*
	*‐*	*‐*	*catII*	*catI*
	*‐*	*‐*	*catIII*	*‐*
Determinant of diaminopyrimidine resistance	*dfrE*	*dfrE*	*dfrE*	*dfrE*
Determinant of triclosan resistance	*MexR*	*MexR*	*MexR*	*MexR*
Antibiotic inactivation enzyme	*catB3*	*‐*	*‐*	*‐*
	*‐*		*catII*	*catII*
	*‐*	*mphD*	*‐*	*‐*
	*‐*	*mphG*	*‐*	*‐*
	*OXA‐78*	*OXA‐78*	*OXA‐78*	*OXA‐78*
	*tetX*			
	*VIM‐2*	*VIM‐2*	*VIM‐2*	*VIM‐2*
Antibiotic target replacement protein	*sul1*	*‐*	*‐*	*‐*
	*sul2*	*‐*	*‐*	*‐*
	*sul3*	*‐*	*‐*	*‐*
Determinant of fluoroquinolone resistance	*oqxB*	*oqxB*	*oqxB*	*oqxB*
	*rpsJ*	*rpsJ*	*rpsJ*	*rpsJ*
	*tet(35)*	*tet(35)*	*tet(35)*	*tet(35)*
	*tetB(48)*	*tetB(48)*	*tetB(48)*	*tetB(48)*
Determinant of beta‐lactam resistance	*OXA‐209*	*‐*	*‐*	*‐*
	*OXA‐347*	*‐*	*‐*	*‐*
	*TLA‐3*	*TLA‐3*	*TLA‐3*	*TLA‐3*

^‐^not predicted

Overall, more resistance genes were predicted in the fully sequenced clinically pathogenic PR63039 genome than in the other three partially sequenced strains, such as, *cat* gene variant *catB2*,* catB3*,* catB6*,* catB*7, *catB*8, *catB9*, and *catB*10, tetracycline resistance gene *tetX*, sulfonamide resistance gene *sul1*,* sul2*, and *sul3*, and β‐lactam resistance gene *OXA‐209*,* OXA‐347*. Moreover, in PR63039 genome, the resistance genes *tetX*,* cat*,* OXA‐347*, and *OXA‐209* were clustered in an approximately 6 kb region, called MY63039‐RR (Hu et al., 2017). No similar resistance gene cluster could be identified in the genomes of other three clinically pathogenic *M. odoratimimus* strains.

Among CCUG12901, CIP101113, and CCUG10230, the identified resistance genes were almost similar. By comparing with PR63039 genome, CCUG12901 genome contained *mefC* (efflux pump conferring antibiotic resistance) and antibiotic inactivation enzyme (*mphD* and *mphG*), but lacked the resistance gene *cat*. The gene *catII*,* catIII* in CCUG10230, and *catI* in CIP101113 were also not predicted in PR63039 genome.

### 3.8 Virulence factors in the genomes of three clinically pathogenic and three environmental *M. odoratimimus* strains

With the help of VFDB protein Set B database (Chen et al., 2012, 2016) and more stringent cutoff parameters, we obtained the VF genes in the genome of three clinically pathogenic (CCUG.12901, CCUG.10230, CIP.101113) and three environmental (H1bi, L41, CCUG.12700) *M. odoratimimus* isolates (Table 4).

**Table 4:** The VFs predicted in three clinically pathogenic and three environmental *M. odoratimimus* genomes

Classification	Definition	Genes coding for virulence factors					
		Clinically pathogenic *M. odoratimimus*			Environmental *M. odoratimimus*		
		CCUG. 12901	CCUG. 10230	CIP. 101113	H1bi	L41	CCUG. 12700
Capsular polysaccharide	UDP‐N‐acetyl‐D‐galactosamine 6‐dehydrogenase	*capL* (*hasB2*)	*capL*	*‐*	*capL*	*‐*	*capL*
	UDP‐glucose 4‐epimerase	***galE***	***galE***	***galE***	***galE***	***galE***	***galE***
	Bifunctional UDP‐N‐acetylglucosamine pyrophosphorylase/glucosamine‐1‐phosphate N‐acetyltransferase	***glmU***	***glmU***	***glmU***	***glmU***	***glmU***	*‐*
	UDP‐glucose 6‐dehydrogenase	***ugd***	***ugd***	***ugd***	*‐*	***ugd***	*‐*
Capsule sialic acid	UDP‐N‐acetylglucosamine 2‐epimerase	*wbjD/wecB*	*wbjD/wecB*	*wbjD/wecB*	*wbjD/wecB*	*‐*	*wbjD/wecB*
Cell wall Peptidoglycan	Undecaprenyl diphosphate synthase	***uppS***	***uppS***	***uppS***	***uppS***	***uppS***	***uppS***
	Glucose‐1‐phosphate thymidylytransferase	***rmlA***	***rmlA***	***rmlA***	***rmlA***	***rmlA***	***rmlA***
	dTDP‐glucose 4,6‐dehydratase	***rmlB***	***rmlB***	***rmlB***	***rmlB***	*‐*	*‐*
	UDP‐N‐acetyl‐D‐mannosaminuronic acid dehydrogenase	*wecC*	*wecC*	*wecC*	*‐*	*‐*	*wecC*
Intracellular survival factors	Catalase katA	***katA***	***katA***	***katA***	***katA***	***katA***	***katA***
	ATP‐dependent Clp protease proteolytic subunit	***clpP***	***clpP***	***clpP***	***clpP***	***clpP***	***clpP***
	Elongation factor Tu	***EF‐Tu***	***EF‐Tu***	***EF‐Tu***	***EF‐Tu***	***EF‐Tu***	***EF‐Tu***
	Superoxide dismutase	***sodB***	***SodB***	***sodB***	***sodB***	***sodB***	***sodB***
Molecular chaperones	CT396 molecular chaperone DnaK	***DnaK***	***DnaK***	***DnaK***	***DnaK***	***DnaK***	***DnaK***
	60k heat‐shock protein HtpB	***Hsp60***	***Hsp60***	***Hsp60***	***Hsp60***	***Hsp60***	***Hsp60***
Urease	Urease	***ureA***	***ureA***	***ureA***	***ureA***	***ureA***	***ureA***
	Urease	***ureB***	***ureB***	***ureB***	***ureB***	***ureB***	***ureB***
	Urease/hydrogenase‐associated predicted GTPase	***ureG***	***ureG***	***ureG***	***ureG***	***ureG***	***ureG***
Acinetobactin	ABC‐type enterochelin transport system, ATPase component	***bauE***	***bauE***	***bauE***	***bauE***	*‐*	***bauE***
Streptococcal enolase	Streptococcal enolase	***eno***	***eno***	***eno***	***eno***	***eno***	***eno***
Pantothenate synthesis	Aspartate 1‐decarboxylase	*panD*	*panD*	*panD*	*panD*	*panD*	*panD*
Heme biosynthesis	Porphobilinogen synthase	*hemB*	*hemB*	*hemB*	*hemB*	*hemB*	*hemB*
	glutamate‐1‐semialdehyde aminotransferase	*hemL*	*hemL*	*hemL*	*hemL*	*hemL*	*hemL*
	Acyl carrier protein	*acpXL*	*acpXL*	*acpXL*	*acpXL*	*acpXL*	*acpXL*
T4SS effectors	Trans‐2‐enoyl‐CoA reductase (no unique name)	***+***	***+***	***+***	***+***	***+***	***+***

^*‐*^not predicted; ^+^,predicted; bold/italic, were discussed.

Overall, all these *M. odoratimimus* genomes had similar VF profiles with a little difference. The VFs included capsule/capsular polysaccharide (*GalE*,* GlmU*,* wbjD/wecB, ugd, uppS, RmlA, RmlB, capL, wecC*), intracellular survival and invasion factors (*katA, clpP, EF‐Tu*,* sodB*), molecular chaperone (*hsp60, DnaK*), urease (*ureA*,* ureB*,* ureG*), acinetobactin (*bauE*), *Streptococcal* enolase (*eno*), heme biosynthesis (*hemB*,* hemL*), acyl carrier protein (*acpXL*), and T4SS effectors (Trans‐2‐enoyl‐CoA reductase).

## 4 DISCUSSION

### 4.1 Genomic evolution, variants, synteny of *M. odoratimimus*

To some contents, the 10 *Myroides* genomes had similar general features. However, the genomes of four clinically pathogenic *M. odoratimimus* strains (PR63039, CCUG12901, CCUG10230, and CIP101113) contained highly variable regions. The genome arrangement, rearrangements of the four clinically pathogenic isolates, and the three environmental strains were similar, respectively. These data implied that they might be evolutionarily related. All four clinically pathogenic isolate genomes contained prophage elements. CRISPRs were not always identified in all the genomes of four clinically pathogenic and three environmental *M. odoratimimus* strains. Complete genomic sequencing of these *M. odoratimimus* strains and their plasmids are indispensable for confirming these analyses.

### 4.2 Resistance genes in clinically pathogenic *M. odoratimimus*

All these four clinically pathogenic strains (PR63039, CCUG12901, CCUG10230, CIP101113) contained a number of antibiotic resistance genes. PR63039 genome might have several possibly unique resistance genes, including *catB2*,* catB3*,* catB6*,* catB7*,* catB8*,* catB9*,* catB*10, *tetX*,* OXA‐209*,* OXA‐347*,* sul1*,* sul2*, and *sul3*. Some were already discussed to be involved in drug resistance in our previous report (Hu et al., 2017). Here, we mainly discuss *mph* and *cat* genes (*catB2*,* catB3*,* catB6*,* catB8*) with clear functions in antibiotic resistance described by literatures.

The *mphD* and *mphG* genes provide a high level of resistance to 14‐ and 15‐membered‐ring macrolides via coding for a macrolide 2′‐phosphotransferase (https://card.mcmaster.ca/). *Cat* (chloramphenicol acetyltransferase gene) has many variants in a variety of bacteria, such as *Staphylococcus aureus*,* Staphylococcus haemolyticus*,* Enterococcus faecium*, and *Bacillus clausii* (Bruckner & Matzura, 1985; Galopin, Cattoir, & Leclercq, 2009; Grady & Hayes, 2003; Schwarz & Cardoso, 1991). All the above identified *catB* genes (as well catI, catII, catIII) are plasmid, chromosome, or integron‐mediated *cat* variants (https://card.mcmaster.ca/). The *cat* variants usually participate in the composition of gene cassette or integron, and confer to the ability of antibiotic resistance. For instance, *catB2*,* aacC4*, and *aadA1* form the gene cassettes aacC4‐aadA1‐catB2, which confers multidrug and broad‐spectrum cephalosporin resistance in *Salmonella* clinical isolates (Villa et al., 2002). *CatB3*, one member of gene cassette aacA7‐catB3‐aadB‐oxa2‐orfD, can be mobilized by the integron‐encoded DNA integrase and plays a role in chloramphenicol resistance of plasmid pBWH301 (Bunny, Hall, & Stokes, 1995; Houang, Chu, Lo, Chu, & Cheng, 2003). *CatB6*, a chloramphenicol acetyltransferase‐encoding allele of the *catB* family inserted in integron In31, functions to decrease the in vitro antibiotic susceptibilities of *Pseudomonas aeruginosa* strains (Laraki et al., 1999). *CatB8* consists of a resistance gene cassette aacA4‐catB8‐aadA1 which is prevalent in many clinical antibiotic‐resistant bacteria, such as carbapenem‐resistant *Klebsiella pneumoniae* (Ou, Li, Li, & Yu, 2017) and carbapenem‐resistant *Acinetobacter baumannii* (Farshadzadeh et al., 2015; Lin, Liou, Tu, Yeh, & Lan, 2013).

We could not further correlate these predicted antibiotic resistance gene profiles to the antibiotic susceptibility of the other three clinically pathogenic strains (CCUG12901, CCUG10230, CIP101113) due to the lack of these related data. However, the conservation of the antibiotic resistance genes among the clinically pathogenic *M. odoratimimus* strains indicated that these predicted antibiotic resistance gene profiles potentially provide the guidance for treating *M. odoratimimus* infections later.

### 4.3 Pathogenicity of clinically pathogenic and environmental *M. odoratimimus* indicated by the predicted virulence factors

The VFs in *M. odoratimimus* were identified using VFDB protein Set B database which curates the experiment‐verified and predicted virulence factor genes (Table 4). The finding of similar VFs in both clinically pathogenic and environmental *M. odoratimimus* genomes might explain the pathogenicity of the three clinically pathogenic *M. odoratimimus* isolates and indicate that the three environmental *M. odoratimimus* isolates are also potentially pathogenic. We only discuss the experimentally verified VF genes (in bold/italic, Table 4).

*bauE* encodes the ferric siderophore ABC transporter/ATP‐binding protein BauE with a high‐affinity iron‐chelating capacity, belonging to acinetobactin. In a mouse sepsis model, the expression of a fully active acinetobactin‐mediated iron uptake apparatus by *Acinetobacter baumannii* was verified to be vital for the bacteria to establish infection and kill mouse, by competing with host cells for iron (Gaddy et al., 2012). Thus, *bauE* should be a survival factor of *M. odoratimimus* during infection.

Capsular *LPS* is the predominant virulence determinant in many gram‐negative and ‐positive bacteria (García & López, 1997). It inhibits complement‐mediated lysis, phagocytosis, and immune recognition in host (Rowe & Huntley, 2015). Several genes involved in capsular *LPS* biosynthesis pathway were found in the *M. odoratimimus genomes*, such as *GalE*,* GlmU*,* ugd, wbjD/wecB, uppS, RmlA, RmlB*. UDP‐sugar 4‐epimerase (GalE) plays an essential role in LPS synthesis (Beerens, Soetaert, & Desmet, 2015; Fry et al., 2000) by catalyzing the interconversion of UDP‐galactose and UDP‐glucose (Fry et al., 2000), is a critical virulence factor in many gram‐negative bacteria (Beerens et al., 2015; Fry et al., 2000). N‐acetylglucosamine‐1‐phosphateuridyltransferase/glucosamine‐1‐phosphate‐acetyltransferase (GlmU) is involved in the synthesis of peptidoglycan and *LPS* in Gram‐negative and ‐positive bacteria (Sharma & Khan, 2017). The bifunctional enzyme UDP‐N‐acetylglucosamine 2‐epimerase/ManNAc kinase (encoded by *wbjD/wecB,* also known as *siaA* or *neuC*) catalyzes biosynthesis of *Escherichia coli* K1 capsule, an alpha‐2,8‐linked polymer of sialic acid, and is a vital meningitis virulence factor for this pathogen (Vann et al., 2004). Both mammals (Chou, Hinderlich, Reutter, & Tanner, 2003) and bacteria (Murkin, Chou, Wakarchuk, & Tanner, 2004) produce this bifunctional enzyme. Both source of this enzyme can catalyze the conversion of UDP‐GlcNAc into ManNAc and UDP, the first two steps in the sialic acid biosynthesis in mammals (Chou et al., 2003) and the first step of sialic acid (N‐acetylneuraminic acid) biosynthesis in bacteria (Murkin et al., 2004). UDP‐glucose 6‐dehydrogenase (UGD) has an indispensable role in hyaluronic acid capsule production and pathogenicity in Group A *Streptococcus* (Cole et al., 2012), is required for bacterial growth inside macrophages (Mouslim & Groisman, 2003). Undecaprenyl diphosphate synthase (UPPS) is involved in cell wall biosynthesis (peptidoglycan and wall teichoic acid synthesis) by catalyzing the synthesis of a polyisoprenoid, becoming an attractive antibacterial drug target (Farha et al., 2015). Glucose‐1‐phosphate thymidylyltransferase (RmlA) is vital for bacteria survival (Mansuri et al., 2016). It participates in L‐rhamnose synthesis (Alphey et al., 2013; Mansuri et al., 2016), a critical linker of peptidoglycan and arabinogalacton in bacterial cell wall (Mansuri et al., 2016), by catalyzing the generation of dTDP‐D‐glucose and pyrophosphate (PPi) (Alphey et al., 2013; Mansuri et al., 2016). dTDP‐D‐glucose 4,6‐dehydratase (RmlB) is also involved in L‐rhamnose biosynthesis, by catalyzing the conversion of dTDP‐D‐glucose into dTDP‐4‐keto‐6‐deoxy‐D‐glucose in cell wall (Allard et al., 2002). Bacteria with truncated *LPS* molecules due to the L‐rhamnose synthesis failure could not prevent clearance by the host cells and become avirulent (Allard et al., 2002). The presence of capsular *LPS* biosynthesis genes in six *M. odoratimimus* indicates their infectivity. The presence of these *LPS* biosynthesis genes in both the three clinically pathogenic and the three environmental *M. odoratimimus* isolates is in concert with the fact that *M. odoratimimus* is gram‐negative and confer it this bacterium with in infectivity.

The presence of bacterial intracellular survival factors *katA, clpP, EF‐Tu,* and *sodB* in the six *M. odoratimimus* genomes suggested that this bacterium might be able to survive within host cells, increase the antibiotic therapy difficulty and thus explain the reported high death rate of *M. odoratimimus* infections (summarized in Hu et al., 2016). Catalase *katA* is a critical virulence factor for *Campylobacter jejuni*, a facultatively intracellular microbe and the principal pathogen of human gastroenteritis. Its resistance ability to the bacteriocidal activity from host cell‐produced hydrogen peroxide and intramacrophage persistence/growth is dependent on this catalase (Day, Sajecki, Pitts, & Joens, 2000). *ClpP*, a highly conserved protease in prokaryotes and eukaryotes, is involved in the rapid adaption capacity during infection for *Listeria monocytogenes*, another facultative intracellular pathogen (Gaillot, Bregenholt, Jaubert, Di Santo, & Berche, 2001; Gaillot, Pellegrini, Bregenholt, Nair, & Berche, 2000). By interacting with host surface nucleolin, the bacterial surface *EF‐Tu* (elongation factor Tu), a GTP‐binding protein involved in protein translation in *Francisella tularensis*, a highly infectious intracellular gram‐negative bacterium, plays a crucial role in its invasion to host tissues (Barel et al., 2008). It is also an adhesion/invasion factor secreted by microbes during infection by bacteria (like Helicobacter pylori) (Chiu, Wang, Tsai, Lei, & Liao, 2017) and fungi (Marcos et al., 2016) through binding (*Mycobacterium avium subsp. paratuberculosis*) with fibronectin on host cells (Viale et al., 2014). Superoxide dismutases (SODs) protect the bacteria from oxidative damage by converting endogenously generated superoxide radicals into hydrogen peroxide and oxygen, are indispensable for intraphagocytic viability for pathogenic bacteria (Dhar, Gupta, & Virdi, 2013), *SodB* is required for colonization of *Helicobacter pylori* in the stomach (Tsugawa et al., 2015).

The presence of bacterial *DnaK* (known as Hsp70 in eukaryotes) and Hsp60 in *M. odoratimimus* imply that the autoimmunological response might be complicated by the infection. Heat‐shock proteins are ubiquitous proteins with high homology between eukaryotes and prokaryotes. Bacterial *DnaK* is crucial bacterial virulence factor. Both host Hsp70 and bacterial *DnaK* mediate bacterial attachment to host cells. After infection, bacterial *DnaK* switches on bacterial survival processes and arouses autoimmune sequelae (Ghazaei, 2017). Hsp60 is also involved in *Clostridium difficile* attachment to host cells (Hennequin et al., 2001), and the strong proinflammatory reaction (IL8) of monocytic cells induced by *Helicobacter pylori *(Lin et al., 2005). *Chlamydia pneumonia* Hsp60 can help to spread *Chlamydial* infection of blood monocytes to vascular wall cells (Rupp et al., 2005), and increase the pathogenesis and severity of *Chlamydia* infection‐correlated atherosclerosis because of sequence homology between bacterial and human Hsp60 (mitochondria in endothelial cells) and subsequent induction of a strong autologous humoral and cellular immune responses (Kalayoglu et al., 2000; Mehta et al., 2005).

The presence of *ureA*,* ureB*,* ureG* in *M. odoratimimus* implies that this bacterium might be pathogenic in human stomach. *Urease* is a principal virulence factor of human gastric bacterium *Helicobacter pylori* (Stingl et al., 2008). It has oligomeric Ni^2+^‐containing heterodimer of *UreA* and *UreB* subunits involved in converting gastric juice urea into NH_3_ in bacterial periplasm which maintains an optimal pH, inner membrane potential and proton motive force, being critical for colonization within the human stomach (Sachs, Weeks, Melchers, & Scott, 2003). *Urease* activity needs an assembly of a lysine‐carbamate functional group with two Ni^2+^ ions facilitated partially by GTP hydrolysis by UreG (Martin‐Diaconescu, Bellucci, Musiani, Ciurli, & Maroney, 2012; Zambelli, Turano, Musiani, Neyroz, & Ciurli, 2009).

The surface enolase (*eno*) of bacteria, a glycolytic pathway enzyme, can bind human plasminogen and convert it into active plasmin (Cork et al., 2009) to facilitate bacterial adherence to host cells and destruction of host tissues through plasmin degrading intercellular junctions and extracellular matrix components (Attali, Durmort, Vernet, & Di Guilmi, 2008), like cellulitis (Bachmeyer et al., 2008), necrotizing fasciitis (Crum‐Cianflone, Matson, & Ballon‐Landa, 2014), and make the bacterial infection life‐threatening (Cork et al., 2009). The containing *eno* in *M. odoratimimus* genome might explain the high death rate of patients infected by *M. odoratimimus* (as summarized in Hu et al., 2016).

In brief, *M. odoratimimus* not only possesses common virulence factors, like using *bauE* gene to compete the iron with host, general *LPS* synthesis genes, adherence factors (*DnaK*,* Hsp60*), but also can survive intracellularly (*katA, clpP, EF‐Tu,* and *sodB*), even in human stomach (*ureA*,* ureB*,* ureG*), but also disseminate easily, destroy human tissues, induce autoimmune diseases. So, the *M. odoratimimus* is a life‐threatening pathogen as reported (summarized in Hu et al., 2016).

## 5 CONCLUSION

The genomic analysis demonstrated that these *M. odoratimimus* isolates are closely related. Our analyses provided some insights in bacterial pathogenicity and antibiotic resistance mechanisms of *M. odoratimimus* and contribute to future development of the therapeutic regimens in *M. odoratimimus* infections.

## COMPLIANCE WITH ETHICAL STANDARDS

This article does not contain any studies with human participants or animals performed by any of the authors.

## CONFLICT OF INTEREST

None declared.

## References

[mbo3634-bib-0001] Allard, S. T. , Beis, K. , Giraud, M. F. , Hegeman, A. D. , Gross, J. W. , Wilmouth, R. C. , … Naismith, J. H. (2002). Toward a structural understanding of the dehydratase mechanism. Structure, 10, 81–92. 10.1016/S0969-2126(01)00694-3 11796113

[mbo3634-bib-0002] Alphey, M. S. , Pirrie, L. , Torrie, L. S. , Boulkeroua, W. A. , Gardiner, M. , Sarkar, A. , … Naismith, J. H. (2013). Allosteric competitive inhibitors of the glucose‐1‐phosphate thymidylyltransferase (RmlA) from *Pseudomonas aeruginosa* . ACS Chemical Biology, 8, 387–396. 10.1021/cb300426u 23138692

[mbo3634-bib-0003] Arras, S. D. , Chua, S. M. , Wizrah, M. S. , Faint, J. A. , Yap, A. S. , & Fraser, J. A. (2016). Targeted genome editing via CRISPR in the pathogen *Cryptococcus neoformans* . PLoS ONE, 11, e0164322 10.1371/journal.pone.0164322 27711143 PMC5053423

[mbo3634-bib-0004] Attali, C. , Durmort, C. , Vernet, T. , & Di Guilmi, A. M. (2008). The interaction of Streptococcus pneumoniae with plasmin mediates transmigration across endothelial and epithelial monolayers by intercellular junction cleavage. Infection and Immunity, 76, 5350–5356. 10.1128/IAI.00184-08 18725422 PMC2573366

[mbo3634-bib-0005] Bachmeyer, C. , Entressengle, H. , Khosrotehrani, K. , Goldman, G. , Delisle, F. , Arlet, G. , & Grateau, G. (2008). Cellulitis due to *Myroides odoratimimus* in a patient with alcoholic cirrhosis. Clinical and Experimental Dermatology, 33, 97–98. 18039344 10.1111/j.1365-2230.2007.02590.x

[mbo3634-bib-0006] Barel, M. , Hovanessian, A. G. , Meibom, K. , Briand, J. P. , Dupuis, M. , & Charbit, A. (2008). A novel receptor‐ligand pathway for entry of *Francisella tularensis* in monocyte‐like THP‐1 cells: Interaction between surface nucleolin and bacterial elongation factor Tu. BMC Microbiology, 8, 145 10.1186/1471-2180-8-145 18789156 PMC2551611

[mbo3634-bib-0007] Beerens, K. , Soetaert, W. , & Desmet, T. (2015). UDP‐hexose 4‐epimerases: A view on structure, mechanism and substrate specificity. Carbohydrate Research, 414, 8–14. 10.1016/j.carres.2015.06.006 26162744

[mbo3634-bib-0008] Belloir, L. , Billy, P. A. , Hentgen, C. , Fille, A. , & Barrans, A. (2016). *Myroides odoratimimus* bacteremia. Medecine at Maladies Infectieuses, 46, 396–397.[Article in French] 10.1016/j.medmal.2016.05.003 27292170

[mbo3634-bib-0009] Bertels, F. , Silander, O. K. , Pachkov, M. , Rainey, P. B. , & van Nimwegen, E. (2014). Automated reconstruction of whole‐genome phylogenies from short‐sequence reads. Molecular Biology and Evolution, 31, 1077–1088. 10.1093/molbev/msu088 24600054 PMC3995342

[mbo3634-bib-0010] Bland, C. , Ramsey, T. L. , Sabree, F. , Lowe, M. , Brown, K. , Kyrpides, N. C. , & Hugenholtz, P. (2007). CRISPR recognition tool (CRT): A tool for automatic detection of clustered regularly interspaced palindromic repeats. BMC Bioinformatics, 8, 209 10.1186/1471-2105-8-209 17577412 PMC1924867

[mbo3634-bib-0011] Bruckner, R. , & Matzura, H. (1985). Regulation of the inducible chloramphenicol acetyltransferase gene of the *Staphylococcus aureus* plasmid pUB112. The EMBO Journal, 4, 2295–2300. 3865770 10.1002/j.1460-2075.1985.tb03929.x PMC554500

[mbo3634-bib-0012] Bunny, K. L. , Hall, R. M. , & Stokes, H. W. (1995). New mobile gene cassettes containing an aminoglycoside resistance gene, aacA7, and a chloramphenicol resistance gene, catB3, in an integron in pBWH301. Antimicrobial Agents and Chemotherapy, 39, 686–693. 10.1128/AAC.39.3.686 7793874 PMC162606

[mbo3634-bib-0013] Burghartz, M. , Bunk, B. , Sproer, C. , Voget, S. , Daniel, R. , Overmann, J. , … Jahn, M. (2015). Complete genome sequence of the urethral catheter isolate *Myroides sp*. A21. Genome Announcements, 3, e00068–15. 10.1128/genomeA.00068-15 25745004 PMC4358391

[mbo3634-bib-0014] Chen, L. , Xiong, Z. , Sun, L. , Yang, J. , & Jin, Q. (2012). VFDB 2012 update: Toward the genetic diversity and molecular evolution of bacterial virulence factors. Nucleic Acids Research, 40, D641–D645. 10.1093/nar/gkr989 22067448 PMC3245122

[mbo3634-bib-0015] Chen, L. , Zheng, D. , Liu, B. , Yang, J. , & Jin, Q. (2016). VFDB 2016: Hierarchical and refined dataset for big data analysis‐10 years on. Nucleic Acids Research, 44, D694–D697. 10.1093/nar/gkv1239 26578559 PMC4702877

[mbo3634-bib-0016] Chiu, K. H. , Wang, L. H. , Tsai, T. T. , Lei, H. Y. , & Liao, P. C. (2017). Secretomic analysis of host‐pathogen interactions reveals that elongation factor‐Tu is a potential adherence factor of *Helicobacter pylori* during pathogenesis. Journal of Proteome Research, 16, 264–273. 10.1021/acs.jproteome.6b00584 27764940

[mbo3634-bib-0017] Chou, W. K. , Hinderlich, S. , Reutter, W. , & Tanner, M. E. (2003). Sialic acid biosynthesis: Stereochemistry and mechanism of the reaction catalyzed by the mammalian UDP‐N‐acetylglucosamine 2‐epimerase. Journal of the American Chemical Society, 125, 2455–2461. 10.1021/ja021309g 12603133

[mbo3634-bib-0018] Cole, J. N. , Aziz, R. K. , Kuipers, K. , Timmer, A. M. , Nizet, V. , & van Sorge, N. M. (2012). A conserved UDP‐glucose dehydrogenase encoded outside the hasABC operon contributes to capsule biogenesis in group A *Streptococcus* . Journal of Bacteriology, 194, 6154–6161. 10.1128/JB.01317-12 22961854 PMC3486382

[mbo3634-bib-0019] Cork, A. J. , Jergic, S. , Hammerschmidt, S. , Kobe, B. , Pancholi, V. , Benesch, J. L. , … Walker, M. J. (2009). Defining the structural basis of human plasminogen binding by streptococcal surface enolase. The Journal of Biological Chemistry, 284, 17129–17137. 10.1074/jbc.M109.004317 19363026 PMC2719351

[mbo3634-bib-0020] Crum‐Cianflone, N. F. , Matson, R. W. , & Ballon‐Landa, G. (2014). Fatal case of necrotizing fasciitis due to *Myroides odoratus* . Infection, 42, 931–935. 10.1007/s15010-014-0626-0 24806817

[mbo3634-bib-0021] Darling, A. E. , Mau, B. , & Perna, N. T. (2010). progressiveMauve: Multiple genome alignment with gene gain, loss and rearrangement. PLoS ONE, 5, e11147 10.1371/journal.pone.0011147 20593022 PMC2892488

[mbo3634-bib-0022] Day, W. A. Jr , Sajecki, J. L. , Pitts, T. M. , & Joens, L. A. (2000). Role of catalase in *Campylobacter jejuni* intracellular survival. Infection and Immunity, 68, 6337–6345. 10.1128/IAI.68.11.6337-6345.2000 11035743 PMC97717

[mbo3634-bib-0023] Dhar, M. S. , Gupta, V. , & Virdi, J. S. (2013). Detection, distribution and characterization of novel superoxide dismutases from *Yersinia enterocolitica,* Biovar 1A. PLoS ONE, 8, e63919 10.1371/journal.pone.0063919 23704955 PMC3660340

[mbo3634-bib-0024] Farha, M. A. , Czarny, T. L. , Myers, C. L. , Worrall, L. J. , French, S. , Conrady, D. G. , … Brown, E. D. (2015). Antagonism screen for inhibitors of bacterial cell wall biogenesis uncovers an inhibitor of undecaprenyl diphosphate synthase. Proceedings of the National Academy of Sciences of the United States of America, 112, 11048–11053. 10.1073/pnas.1511751112 26283394 PMC4568241

[mbo3634-bib-0025] Farshadzadeh, Z. , Hashemi, F. B. , Rahimi, S. , Pourakbari, B. , Esmaeili, D. , … Bahador, A. (2015). Wide distribution of carbapenem resistant *Acinetobacter baumannii* in burns patients in Iran. Frontier Microbiology, 6, 1146. 10.3389/fmicb.2015.01146 PMC4611150 26539176

[mbo3634-bib-0026] Fry, B. N. , Feng, S. , Chen, Y. Y. , Newell, D. G. , Coloe, P. J. , & Korolik, V. (2000). The galE gene of *Campylobacter jejuni* is involved in lipopolysaccharide synthesis and virulence. Infection and Immunity, 68, 2594–2601. 10.1128/IAI.68.5.2594-2601.2000 10768949 PMC97464

[mbo3634-bib-0027] Gaddy, J. A. , Arivett, B. A. , Mcconnell, M. J. , Lopez‐Rojas, R. , Pachon, J. , & Actis, L. A. (2012). Role of acinetobactin‐mediated iron acquisition functions in the interaction of *Acinetobacter baumannii* strain ATCC 19606T with human lung epithelial cells, *Galleria mellonella* caterpillars, and mice. Infection and Immunity, 80, 1015–1024. 10.1128/IAI.06279-11 22232188 PMC3294665

[mbo3634-bib-0028] Gaillot, O. , Bregenholt, S. , Jaubert, F. , Di Santo, J. P. , & Berche, P. (2001). Stress‐induced ClpP serine protease of *Listeria monocytogenes* is essential for induction of listeriolysin O‐dependent protective immunity. Infection and Immunity, 69, 4938–4943. 10.1128/IAI.69.8.4938-4943.2001 11447171 PMC98585

[mbo3634-bib-0029] Gaillot, O. , Pellegrini, E. , Bregenholt, S. , Nair, S. , & Berche, P. (2000). The ClpP serine protease is essential for the intracellular parasitism and virulence of *Listeria monocytogenes* . Molecular Microbiology, 35, 1286–1294. 10.1046/j.1365-2958.2000.01773.x 10760131

[mbo3634-bib-0030] Galopin, S. , Cattoir, V. , & Leclercq, R. (2009). A chromosomal chloramphenicol acetyltransferase determinant from a probiotic strain of *Bacillus clausii* . FEMS Microbiology Letters, 296, 185–189. 10.1111/j.1574-6968.2009.01633.x 19459958

[mbo3634-bib-0031] García, E. , & López, R. (1997). Molecular biology of the capsular genes of *Streptococcus pneumoniae* . FEMS Microbiology Letter, 49, 1–10. 10.1016/S0378-1097(97)00026-8 9103971

[mbo3634-bib-0032] Ghazaei, C. (2017). Role and mechanism of the Hsp70 molecular chaperone machines in bacterial pathogens. Journal of Medical Microbiology, 66, 259–265. 10.1099/jmm.0.000429 28086078

[mbo3634-bib-0033] Grady, R. , & Hayes, F. (2003). Axe‐Txe, a broad‐spectrum proteic toxin‐antitoxin system specified by a multidrug‐resistant, clinical isolate of *Enterococcus faecium* . Molecular Microbiology, 47, 1419–1432. 10.1046/j.1365-2958.2003.03387.x 12603745

[mbo3634-bib-0034] Grant, J. R. , & Stothard, P. (2008). The CGView Server: A comparative genomics tool for circular genomes. Nucleic Acids Research, 36, W181–W184. 10.1093/nar/gkn179 18411202 PMC2447734

[mbo3634-bib-0035] Hatoum‐Aslan, A. , & Marraffini, L. A. (2014). Impact of CRISPR immunity on the emergence and virulence of bacterial pathogens. Current Opinion in Microbiology, 17, 82–90. 10.1016/j.mib.2013.12.001 24581697 PMC3942673

[mbo3634-bib-0036] Hennequin, C. , Porcheray, F. , Waligora‐Dupriet, A. , Collignon, A. , Barc, M. , Bourlioux, P. , & Karjalainen, T. (2001). GroEL (Hsp60) of *Clostridium difficile* is involved in cell adherence. Microbiology, 147(Pt 1), 87–96. 10.1099/00221287-147-1-87 11160803

[mbo3634-bib-0037] Heritier, C. , Poirel, L. , & Nordmann, P. (2006). Cephalosporinase over‐expression resulting from insertion of ISAba1 in *Acinetobacter baumannii* . Clinical Microbiology and Infection, 12, 123–130. 10.1111/j.1469-0691.2005.01320.x 16441449

[mbo3634-bib-0038] Higgins, P. G. , Dammhayn, C. , Hackel, M. , & Seifert, H. (2010). Global spread of carbapenem‐resistant *Acinetobacter baumanni*i. Journal of Antimicrobial Chemotherapy, 65, 233–238. 10.1093/jac/dkp428 19996144

[mbo3634-bib-0039] Houang, E. T. , Chu, Y. W. , Lo, W. S. , Chu, K. Y. , & Cheng, A. F. (2003). Epidemiology of rifampin ADP‐ribosyltransferase (arr‐2) and metallo‐beta‐lactamase (blaIMP‐4) gene cassettes in class 1 integrons in *Acinetobacter* strains isolated from blood cultures in 1997 to 2000. Antimicrobial Agents and Chemotherapy, 47, 1382–1390. 10.1128/AAC.47.4.1382-1390.2003 12654674 PMC152494

[mbo3634-bib-0040] Hu, S. , Jiang, T. , Zhou, Y. , Ming, D. , Gao, H. , & Wang, M. (2017). Genomic analysis of the multi‐drug‐resistant clinical isolate *Myroides odoratimimus* PR63039. Molecular Genetics and Genomics, 292, 133–144. 10.1007/s00438-016-1261-5 27796642

[mbo3634-bib-0041] Hu, S. H. , Yuan, S. X. , Qu, H. , Jiang, T. , Zhou, Y. J. , Wang, M. X. , & Ming, D. S. (2016). Antibiotic resistance mechanisms of *Myroides sp* . Journal of Zhejiang University‐Science B, 17, 188–199. 10.1631/jzus.B1500068 26984839 PMC4794510

[mbo3634-bib-0042] Jia, B. , Raphenya, A. R. , Alcock, B. , Waglechner, N. , Guo, P. , Tsang, K. K. , … McArthur, A. G. (2017). CARD 2017: Expansion and model‐centric curation of the comprehensive antibiotic resistance database. Nucleic Acids Research, 45(D1), D566–D573. 10.1093/nar/gkw1004 27789705 PMC5210516

[mbo3634-bib-0043] Jover‐Sáenz, A. , Pérez‐Villar, F. , & Barcenilla‐Gaite, F. (2016). Severe sepsis caused by infected prosthesis joint due to *Myroides odoratimimus* . Medicina Clinica (Barc), 147, 276–277. [Article in Spanish] 10.1016/j.medcli.2016.04.011 27207242

[mbo3634-bib-0044] Kalayoglu, M. V. , Indrawati , Morrison, R. P. , Morrison, S. G. , Yuan, Y. , & Byrne, G. I. (2000). Chlamydial virulence determinants in atherogenesis: The role of Chlamydial lipopolysaccharide and heat shock protein 60 in macrophage‐lipoprotein interactions. Journal of Infectious Diseases, 181(Suppl 3), S483–S489. 10.1086/315619 10839744

[mbo3634-bib-0045] Ktari, S. , Mnif, B. , Koubaa, M. , Mahjoubi, F. , Ben Jemaa, M. , Mhiri, M. N. , & Hammami, A. (2012). Nosocomial outbreak of *Myroides odoratimimus* urinary tract infection in a Tunisian hospital. The Journal of Hospital Infection, 80, 77–81. 10.1016/j.jhin.2011.09.010 22099498

[mbo3634-bib-0046] Laraki, N. , Galleni, M. , Thamm, I. , Riccio, M. L. , Amicosante, G. , Frere, J. M. , & Rossolini, G. M. (1999). Structure of In31, a blaIMP‐containing *Pseudomonas aeruginosa* integron phyletically related to In5, which carries an unusual array of gene cassettes. Antimicrobial Agents and Chemotherapy, 43, 890–901. 10103196 10.1128/aac.43.4.890 PMC89222

[mbo3634-bib-0047] Lin, M. F. , Liou, M. L. , Tu, C. C. , Yeh, H. W. , & Lan, C. Y. (2013). Molecular epidemiology of integron‐associated antimicrobial gene cassettes in the clinical isolates of *Acinetobacter baumannii* from northern Taiwan. Annals of Laboratory Medicine, 33, 242–247. 10.3343/alm.2013.33.4.242 23826559 PMC3698301

[mbo3634-bib-0048] Lin, S. N. , Ayada, K. , Zhao, Y. , Yokota, K. , Takenaka, R. , Okada, H. , … Oguma, K. (2005). *Helicobacter pylori* heat‐shock protein 60 induces production of the pro‐inflammatory cytokine IL8 in monocytic cells. Journal of Medical Microbiology, 54(Pt 3), 225–233. 10.1099/jmm.0.45871-0 15713605

[mbo3634-bib-0049] Liu, C. J. , Wang, R. , Gong, F. M. , Liu, X. F. , Zheng, H. J. , Luo, Y. Y. , & Li, X. R. (2015). Complete genome sequences and comparative genome analysis of *Lactobacillus plantarum* strain 5‐2 isolated from fermented soybean. Genomics, 106, 404–411. 10.1016/j.ygeno.2015.07.007 26212213

[mbo3634-bib-0050] Mansuri, R. , Ansari, M. Y. , Singh, J. , Rana, S. , Sinha, S. , Sahoo, G. C. , … Das, P. (2016). Computational elucidation of structural basis for ligand binding with *Mycobacterium tuberculosis* glucose‐1‐phosphate thymidylyltransferase (RmlA). Current Pharmaceutical Biotechnology, 17, 1089–1099. 10.2174/1389201017666160909155959 27633891

[mbo3634-bib-0051] Marcos, C. M. , de Oliveira, H. C. , da Silva, J. F. , Assato, P. A. , Yamazaki, D. S. , da Silva, R. A. , & Fusco‐Almeida, A.M . (2016). Identification and characterisation of elongation factor Tu, a novel protein involved in *Paracoccidioides brasiliensis*‐host interaction. FEMS Yeast Research, 16, fow079 10.1093/femsyr/fow079 27634774

[mbo3634-bib-0052] Martin‐Diaconescu, V. , Bellucci, M. , Musiani, F. , Ciurli, S. , & Maroney, M. J. (2012). Unraveling the Helicobacter pylori UreG zinc binding site using X‐ray absorption spectroscopy (XAS) and structural modeling. Journal of Biological Inorganic Chemistry, 17, 353–361. 10.1007/s00775-011-0857-9 22068961 PMC3354701

[mbo3634-bib-0053] Mcarthur, A. G. , Waglechner, N. , Nizam, F. , Yan, A. , Azad, M. A. , Baylay, A. J. , … Wright, G. D. (2013). The comprehensive antibiotic resistance database. Antimicrobial Agents and Chemotherapy, 57, 3348–3357. 10.1128/AAC.00419-13 23650175 PMC3697360

[mbo3634-bib-0054] Mehta, T. A. , Greenman, J. , Ettelaie, C. , Venkatasubramaniam, A. , Chetter, I. C. , & McCollum, P. T. (2005). Heat shock proteins in vascular disease—a review. European Journal of Vascular & Endovascular Surgery, 29, 395–402. 10.1016/j.ejvs.2005.01.005 15749041

[mbo3634-bib-0055] Mouslim, C. , & Groisman, E. A. (2003). Control of the Salmonella ugd gene by three‐two‐component regulatory systems. Molecular Microbiology, 47, 335–344. 10.1046/j.1365-2958.2003.03318.x 12519186

[mbo3634-bib-0056] Murkin, A. S. , Chou, W. K. , Wakarchuk, W. W. , & Tanner, M. E. (2004). Identification and mechanism of a bacterial hydrolyzing UDP‐N‐acetylglucosamine 2‐epimerase. Biochemistry, 43, 14290–14298. 10.1021/bi048606d 15518580

[mbo3634-bib-0057] Ou, Q. , Li, W. , Li, B. , & Yu, C. (2017). Prevalence of carbapenem‐resistant *Klebsiella pneumoniae* (CRKP) and the distribution of Class 1 Integron in their strains isolated from a hospital in Central China. Chinese Medical Sciences Journal, 32, 107–112. 28693691 10.24920/J1001-9294.2017.018

[mbo3634-bib-0058] Pompilio, A. , Galardi, G. , Gherardi, G. , Verginelli, F. , Geminiani, C. , Pilloni, A. P. , & Bonaventura, G. D. (2017). Infection of recurrent calcaneal ulcer caused by a biofilm‐producer *Myroides odoratimimus* strain. Folia Microbiologica (Praha), 2017, 203–207. 10.1007/s12223-017-0552-5 28956275

[mbo3634-bib-0059] Rowe, H. M. , & Huntley, J. F. (2015). From the Outside‐In: The *Francisella tularensis* envelope and virulence. Frontiers in Cellular and Infection Microbiology, 5, 94. 26779445 10.3389/fcimb.2015.00094 PMC4688374

[mbo3634-bib-0060] Rupp, J. , Koch, M. , van Zandbergen, G. , Solbach, W. , Brandt, E. , & Maass, M. (2005). Transmission of *Chlamydia pneumoniae* infection from blood monocytes to vascular cells in a novel transendothelial migration model. FEMS Microbiology Letters, 242, 203–208. 10.1016/j.femsle.2004.11.010 15621438

[mbo3634-bib-0061] Sachs, G. , Weeks, D. L. , Melchers, K. , & Scott, D. R. (2003). The gastric biology of Helicobacter pylori. Annual Review of Physiology, 65, 349–369. 10.1146/annurev.physiol.65.092101.142156 12471160

[mbo3634-bib-0062] Schwarz, S. , & Cardoso, M. (1991). Molecular cloning, purification, and properties of a plasmid‐encoded chloramphenicol acetyltransferase from *Staphylococcus haemolyticus* . Antimicrobial Agents and Chemotherapy, 35, 1277–1283. 10.1128/AAC.35.7.1277 1929282 PMC245158

[mbo3634-bib-0063] Sharma, R. , & Khan, I. A. (2017). Mechanism and potential inhibitors of GlmU: A novel target for antimicrobial drug discovery. Current Drug Targets, 18, 1587–1597. 27138757 10.2174/1389450117666160502152011

[mbo3634-bib-0064] Siguier, P. , Perochon, J. , Lestrade, L. , Mahillon, J. , & Chandler, M. (2006). ISfinder: The reference centre for bacterial insertion sequences. Nucleic Acids Research, 34, D32–D36. 10.1093/nar/gkj014 16381877 PMC1347377

[mbo3634-bib-0065] Sontheimer, E. J. , & Barrangou, R. (2015). The bacterial origins of the CRISPR genome‐editing revolution. Human Gene Therapy, 26, 413–424. 10.1089/hum.2015.091 26078042

[mbo3634-bib-0066] Stingl, K. , Schauer, K. , Ecobichon, C. , Labigne, A. , Lenormand, P. , Rousselle, J. C. , & de Reuse, H. (2008). In vivo interactome of *Helicobacter pylori* urease revealed by tandem affinity purification. Molecular & Cellular Proteomics, 7, 2429–2441. 10.1074/mcp.M800160-MCP200 18682379

[mbo3634-bib-0067] Tsugawa, H. , Mori, H. , Matsuzaki, J. , Masaoka, T. , Hirayama, T. , Nagasawa, H. , & Suzuki, H. (2015). Nordihydroguaiaretic acid disrupts the antioxidant ability of *Helicobacter pylori* through the repression of SodB activity in vitro. BioMed Research International, 2015, 734548. 25945343 10.1155/2015/734548 PMC4402480

[mbo3634-bib-0068] Vann, W. F. , Daines, D. A. , Murkin, A. S. , Tanner, M. E. , Chaffin, D. O. , Rubens, C. E. , … Silver, R. P. (2004). The NeuC protein of *Escherichia coli* K1 is a UDP N‐acetylglucosamine 2‐epimerase. Journal of Bacteriology, 186, 706–712. 10.1128/JB.186.3.706-712.2004 14729696 PMC321479

[mbo3634-bib-0069] Viale, M. N. , Echeverria‐Valencia, G. , Romasanta, P. , Mon, M. L. , Fernandez, M. , Malchiodi, E. , … Santangelo, M. L. (2014). Description of a novel adhesin of Mycobacterium avium subsp. paratuberculosis. BioMed Research International, 2014, 729618. 25136616 10.1155/2014/729618 PMC4130151

[mbo3634-bib-0070] Villa, L. , Mammina, C. , Miriagou, V. , Tzouvelekis, L. S. , Tassios, P. T. , Nastasi, A. , & Carattoli, A. (2002). Multidrug and broad‐spectrum cephalosporin resistance among Salmonella enterica serotype enteritidis clinical isolates in southern Italy. Journal of Clinical Microbiology, 40, 2662–2665. 10.1128/JCM.40.7.2662-2665.2002 12089302 PMC120561

[mbo3634-bib-0071] Willems, P. , Muller, J. , Verhaegen, J. , Saegeman, V. , & Desmet, S. (2017). How to treat a fulminant erysipelas and sepsis caused by *Myroides odoratimimus*: Case report and literature review. Acta Clinica Belgica, 72, 331–335. 10.1080/17843286.2016.1245173 27765000

[mbo3634-bib-0072] Zambelli, B. , Turano, P. , Musiani, F. , Neyroz, P. , & Ciurli, S. (2009). Zn2+‐linked dimerization of UreG from *Helicobacter pylori*, a chaperone involved in nickel trafficking and urease activation. Proteins, 74, 222–239. 10.1002/prot.22205 18767150

[mbo3634-bib-0073] Zhou, Y. , Liang, Y. , Lynch, K. H. , Dennis, J. J. , & Wishart, D. S. (2011). PHAST: A fast phage search tool. Nucleic Acids Research, 39, W347–W352. 10.1093/nar/gkr485 21672955 PMC3125810

