# Supplementary material for: Comparative genomic analysis of Myroides odoratimimus isolates
Source: Microbiologyopen. 2018 May 23;8(2):e00634. doi: 10.1002/mbo3.634 (PMC6391281; doi:10.1002/mbo3.634)
Supplement: Supplementary file 1 [file MBO3-8-e00634-s001.docx]

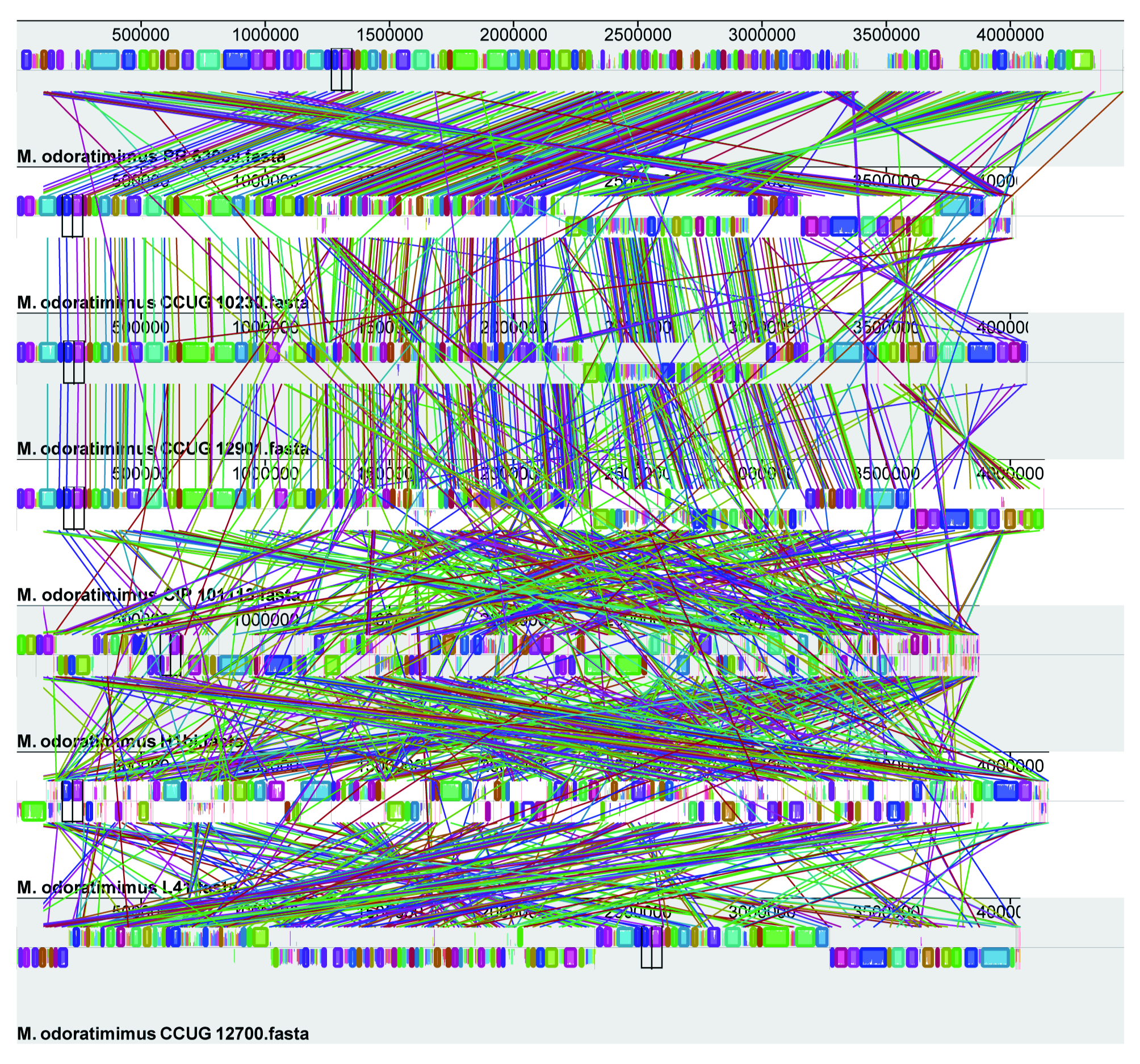


**FIGURE S1 Alignment of** **the genomes of four clinically pathogenic isolates (****PR63039, CCUG10230,** **CCUG12901, CIP101113) and three environmental bacteria (H1bi, L41, CCUG12700) using software progressive MAUVE**. Colored blocks: they surrounded a region of the genome sequence and were presumably homologous and internally free from genomic rearrangement. Regions outside blocks: they lacked detectable homology among the input genomes. Completely white areas: they were not aligned and probably contained sequence elements specific to a particular genome.

**TABLE S1 CRISPRs prediction in the genomes of four clinically pathogenic and three environmental *M. odoratimimus* strains**

| Strains | CRISPRs Sequences | Range |
| --- | --- | --- |
| PR63039 | CTTTTAACTGCACCATATAGGTATTGAAAC | 3841825–3846203 |
|  | CTTCTAATCGCACCATATAGGAAACAAAAA | 3847529–3848812 |
|  | GTTGGTACTGTACACGTATCTGGTGTGCAATCACAAC | 4175953–4176659 |
| CCUG10230 | TTGTGATTGCACACCAGATACGTGTACAGTACCAAC | 2336421–2336925 |
|  | GTTGTGATTGCACACCAAAAACGTGTACAGTACCAAC | 2337132–337302 |
|  | TTTTGTTTCCTATATGGTG | 2673220-2676400 |
|  | NNTTCAATACCTATATGGTGCAGTTAAAAG | 2676510-2679118 |
| CCUG12700 | CTTTTAACCGCATCATATAGGTATTGAAA | 509302–512168 |
| H1bi | CTTCTAATCATACCATATAGGAATTGAAAG | 419709–421974 |
| CCUG12901 | ﹣ | ﹣ |
| CIP101113 | ﹣ | ﹣ |
| L41 | ﹣ | ﹣ |

﹣: not predicted
